# Occurrence of variants of unknown clinical significance in genetic testing for hereditary breast and ovarian cancer syndrome and Lynch syndrome: a literature review and analytical observational retrospective cohort study

**DOI:** 10.1186/s12920-023-01437-7

**Published:** 2023-01-16

**Authors:** Felicia Adam, Muriel Fluri, Amina Scherz, Manuela Rabaglio

**Affiliations:** 1 Medical Faculty of the University of Bern, Bern, Switzerland; 2 Department of Medical Oncology, Inselspital, Bern University Hospital, University of Bern, Bern, Switzerland

**Keywords:** Hereditary breast and ovarian cancer, Lynch syndrome, VUS, *BRCA1*, *BRCA2*, MMR genes, Multigene panel

## Abstract

**Background and purpose:**

Over the last decade, the implementation of multigene panels for hereditary tumor syndrome has increased at our institution (Inselspital, University Hospital Berne, Switzerland). The aim of this study was to determine the prevalence of variants of unknown significance (VUS) in patients with suspected Lynch syndrome and suspected hereditary breast and ovarian cancer syndrome, the latter in connection with the trend toward ordering larger gene panels.

**Results:**

Retrospectively collected data from 1057 patients at our institution showed at least one VUS in 126 different cases (11.9%). In patients undergoing genetic testing for *BRCA1/2*, the prevalence of VUS was 6%. When < 10 additional genes were tested in addition to *BRCA1/2*, the prevalence increased to 13.8%, and 31.8% for > 10 additional genes, respectively. The gene most frequently affected with a VUS was *ATM*. 6% of our patients who were tested for Lynch syndrome had a VUS result in either *MLH1, MSH2* or *MSH6*.

**Conclusions:**

Our data demonstrate that panel testing statistically significantly increases VUS rates due to variants in non-*BRCA* genes. Good genetic counseling before and after obtaining results is therefore particularly important when conducting multigene panels to minimize patient uncertainty due to VUS results.

**Supplementary Information:**

The online version contains supplementary material available at 10.1186/s12920-023-01437-7.

## Background

### Genetic testing

Genetic testing for germline sequence variants is important for assessing the risk of hereditary tumor syndromes [1]. Genetic predisposition for cancer is a complex matter because cancer risk can be increased by pathogenic variants in different genes, and a variant at a particular site may cause more than one type of cancer [2]. Researchers have identified different inherited tumor syndromes and their causative genes. According to these findings, different genes were analyzed depending on the suspected tumor syndrome [3]. Traditionally, testing for specific genes is performed after evaluating various factors such as family history, age of diagnosis and histological type of cancer [4]. This study focuses on hereditary breast and ovarian cancer (HBOC; MIM#114480) and Lynch Syndrome (LS; MIM#120435)/hereditary non-polyposis colorectal cancer (HNPCC).

Regarding HBOC the genes *BRCA1* and *BRCA2* have been known for more than two decades [5–7]. Myriad Genetic Laboratories introduced complete sequence analysis of these genes in 1996 as one of the first sequence-based tests for risk assessment of a common cancer [8]. Today, sequence based tests are omnipresent in genetic testing [9]. The development of next generation sequencing (NGS) technologies has greatly reduced time consumption and cost of simultaneous analysis and thereby increased the use of multigene panels in genetic testing for hereditary tumor syndromes [2, 10]. The National Comprehensive Cancer Network (NCCN) provides guidelines for genetic/familial high-risk assessment of tumor syndromes that include benefits and limitations of multigene testing using NGS [11, 12]. According to these guidelines, multigene testing can be beneficial because a family history of cancer may be explained by more than one cancer syndrome and some individuals may carry pathogenic/likely pathogenic variants in more than one gene. In addition, multigene testing may also include genes with a lower penetrance for a particular cancer syndrome, so-called moderate-risk genes. While this is an advantage of NGS, testing moderate-risk genes also comes with the limitation that some variants found will not alter patient risk management. Another limitation are higher rates of variant without clear clinical significance [11].

In addition to the NCCN guidelines, the following guidelines for genetic testing and counseling are valid in Switzerland and are used in our population: the SAKK Swiss guidelines [13, 14] for breast and ovarian cancer and the PREMM5 Model [15] for Lynch Syndrome.

### Classification of variants

Genetic testing can have different results. Beside the possibilities of a variant being pathogenic (leading to a significantly elevated risk of cancer) or benign (not interfering with the function of the protein), it’s impact on the protein function can also be uncertain. Such an alteration is considered a variant of unknown significance (VUS) [9, 16, 17]. The detailed classification of the variants is shown in Table 1. To avoid misinterpretation when describing the pathogenicity of variants, the Evidence-based Network for the Interpretation of Germline Mutation Alleles (ENIGMA) has established appropriate terminology for describing genetic variants [16]. They propose to use the IARC (International Agency for Research on Cancer) five-tier variant classification system [9] to describe clinical relevance of genetic variant. This is used by ENIGMA for *BRCA1/2* variant classification, and by the International Society for Gastrointestinal Hereditary Tumours (InSIGHT) group for mismatch repair (MMR) gene variant classification [17], as well as by databases used to classify genetic variants such as ClinVar [18]. A very similar probability-based method of variant classification was established by the American College of Medical Genetics and Genomics and the Association for Molecular Pathology (ACMG/AMP) [19]. The clinical implications and proposed predictive tests for relatives at risk largely overlap with the IARC and the ACMG/AMP classifications [16].

**Table 1:** Classification of variants according to ENIGMA [16]

Class	Description	Probability of being Pathogenic		Clinical implication [16]
		According to IARC [9]	According to ACMG/AMP [19]	
1	Benign (B)/ of no clinical significance	< 0.001	< 0.001	Treat as if ‘no mutation detected’ for this disorder No Predictive testing of at risk relatives is suggested
2	Likely benign (LB)/ of little clinical significance	0.001–0.049	0.001–0.099	
3	Variant of unknown significance (VUS)	0.050–0.949	0.100–0.899	Surveillance based on family history and patient-based risk factors Obtaining additional data in order to reclassify, if possible Research testing of family members can be recommended
4	Likely pathogenic (LP)	0.950–0.999	0.900–0.999	Full high-risk surveillance Predictive testing of at-risk relatives is suggested
5	Pathogenic (P)	> 0.999	> 0.999	

In our study, we do not distinguish between pathogenic and likely pathogenic outcomes, but refer to them as P/LP

### Variants of unknown significance

Most VUS are missense mutations, but also include intronic variants and inframe deletions and insertions [20]. There is a growing number of functional assays to test VUS on the protein level [21, 22] but in several cases the effect of a VUS remains unclear. Thus its clinical relevance remains uncertain and should not be used as a sole parameter to guide patient and relatives management [2]. Clinical management recommendations should rather be based on personal and family history of disease [16]. Therefore, VUS results in genetic testing may lead to uncertainty in patients rather than clarity. VUS are also frequently misinterpreted by physicians who order genetic testing [23, 24]. As the number of genes tested is directly related to the occurrence of VUS, the risk of inappropriate recommendations that may harm patients could increase with the use of multigene panel analysis [1, 2]. Nevertheless, studies have shown that through genetic counseling, VUS results do not lead to excessive surgery or overstress, but are only less reassuring than negative test results [25] and that surgical decisions are based heavily on the patient’s context and not just a VUS result [26].

### Hereditary breast and ovarian cancer

*BRCA1* and *BRCA2* are well known genes with a proven association to HBOC [5–7]. The prospective cohort study by Kuchenbaecker et al. [27] showed that the cumulative risk of breast cancer by age 80 years was approximately 72% for *BRCA1* and 69% for *BRCA2* mutation carriers. The corresponding ovarian cancer risks was found to be 44% for *BRCA1* and 17% for *BRCA2* mutation carriers. Therefore, a diagnosis with a pathogenic variant in one of those genes clearly affects the patient, its treatment and prevention from other cancers [28].

Back in 1999, Easton stated in a paper on predisposition genes for breast cancer [29] that only 20–25% of the familial risk for breast cancer is due to susceptibility genes known at that time, including *BRCA1*, *BRCA2*, *TP53*, *PTEN* and *ATM*. More recent studies describe other high or moderately high risk and susceptibility genes of breast [30] and ovarian [31, 32] cancer.

The expected prevalence of pathogenic mutations in *BRCA1*/*BRCA2* genetic testing of patients who meet testing guidelines is about 9% according to previous studies [30, 33, 34]. These and other studies have also shown, that next generation sequencing multigene panels can detect pathogenic variants in susceptibility genes, that would be missed in traditional *BRCA1*/*BRCA2* testing [30, 33–36]. This means that larger gene panels will detect more pathogenic variants than if only the high risk genes *BRCA1* and *BRCA2* are tested. At the same time, the prevalence of VUS is also increasing while testing more and more susceptibility genes.

Several studies attempted to determine the prevalence of VUS in *BRCA1* and *BRCA2* genetic testing, and the results varied widely by population, with an overall VUS-prevalence of 7–15% [37]. With further testing and reclassification of VUS, the detection rate declined to approximately 5–10% in *BRCA* genetic testing conducted in the United States [38]. Myriad Genetic Laboratories [39] recorded a VUS decline from 13% to only 2.1% in 2013. In less well studied genes, the number of VUS remains high [35].

### Lynch syndrome

LS, also known as hereditary nonpolyposis colorectal cancer, is the most common hereditary colorectal cancer type [40]. It is responsible for about 1% of colorectal tumors [41, 42]. LS is caused by heterozygous germline inactivation of the DNA mismatch repair genes (MMR) *MLH1, MSH2, MSH6* and *PMS2* [40]. In addition, Ligtenberg et al. [43] described that a deletion at the 3' end of the gene *EPCAM*, which is located upstream of the *MSH2* gene, can cause epigenetic silencing of the *MSH2* gene and thus also lead to LS. Mutations in MMR genes lead to an accumulation of mutations in microsatellites in tumors, so called microsatellites instability (MSI) [44]. Patients with LS have a very high risk of developing colorectal cancer (25–70%) and endometrial cancer (30–70%), and are also at higher risk of ovarian, gastric, small bowel, pancreatic, urothelial, renal, prostate, breast cancer and central nervous system tumors than the average population [44–49].

### Aims and benefit of the study

In our study, patients genetically tested for one of the hereditary tumor syndromes HBOC or LS are divided into cohorts according to the genetic test performed. In the beginning of genetic testing in the UKMO (University Hospital for Medical Oncology) at the Inselspital, mainly *BRCA1* and *BRCA2* genes were tested for HBOC and DNA mismatch repair genes *MLH1*, *MSH2*, *MSH6* and *PMS2* were tested for LS. While testing for LS has remained the same over the years more multigene panels have been conducted for HBOC since the beginning of 2016.

The aim of this study is to determine the rates of pathogenic variants and VUS in genetic testing and the increase of VUS regarding broader use of multigene panels. We are investigating the hypothesis that the larger the gene panels for HBOC, the higher the incidence of VUS in our patient population. Therefore, the null hypothesis to be refuted is that there is no correlation between the number of genes tested and the occurrence of VUS. At the same time, we do not expect an increase of VUS in the Lynch Syndrome cohort, as the panel did not change over time. In addition, we evaluate in which genes included in commonly used HBOC panels [50, 51] the most variants (P/LP and VUS) occur in our study.

This study evaluates genetic panels currently used in our clinic and compares the outcome to international literature. This contributes to the quality assurance of genetic testing at the UKMO, as the actual rates of P/LP and VUS are used to assess the utility of multi-gene panels [52].

## Methods

### Study design and setting

In this retrospective cohort study the primary aim is to show the prevalence of VUS in our patient population and its increase regarding broader use of gene panels.

### Participant characteristics

Included in the study are patients who underwent genetic testing in the UKMO at the Inselspital between 2010 and 2021. The patient population contains male and female participants over the age of 18 years, who were genetically tested for HBOC or HNPCC, due to a diagnosis with breast, ovarian, endometrial, cervical, fallopian tube, colon cancer and/or several other types of carcinomas or whose family history points to one of these hereditary tumor syndromes (n = 1057).

Patients tested for known familial mutations or germline analysis based on somatically detected variants were excluded from the study, as these cases were not relevant to the research question.

Tests performed for hereditary tumor syndromes other than HBOC or HNPCC (e.g., Li-Fraumeni syndrome and familial adenomatous polyposis) were also excluded.

The indication for test followed national (SAKK) [14] and international guidelines (NCCN) [11] and the costs were covered by the medical insurance and complying with the Swiss law.

### Materials

The different genetic tests and multigene panels used in our study are listed in Table 2. The commissioned tests were carried out in different laboratories in Switzerland, namely Genetica [50] in Zurich, Center for Laboratory Medicine at the Cantonal Hospital Aarau (KSA) [51], Medical Genetics Department at the Valais Hospital [53], Department of Genetic Medicine at the University Hospital of Geneva (HUG) [54] and Institute of Medical Genetics and Pathology at the University Hospital Basel [55]. In most cases, gene panel diagnostics provided by the laboratory were performed. However, the laboratories also offered us the possibility to assemble personalized panels depending on the patient's medical history and pedigree. 38 cases of personalized panels are included in our analyses and assigned to the corresponding cohorts according to the number of genes investigated.

**Table 2:** Panels for hereditary tumor syndromes

Panel	Lab	List of genes	Cohort
*Hereditary breast and ovarian cancer*			
*BRCA* standard	Genetica, KSA, HUG or Valais	*BRCA1* and *BRCA2*	*BRCA1*/2 = 1
*BRCA* plus Mamma/Breast cancer panel	Genetica, KSA	*BRCA1, BRCA2, ATM, CDH1, CHEK2, PALB2, PTEN, STK11, TP53*	*BRCA* plus ≤ 10 genes = 2
*BRCA* plus Ovar/Ovarian cancer panel	Genetica, KSA	*BRCA1, BRCA2, BRIP1, EPCAM (3’UTR), MLH1, MSH2, MSH6, PMS2, RAD51C, RAD51D*	2
Customized		*BRCA* plus ≤ 10	2
*BRCA* plus Lynch	Genetica, KSA	*BRCA1, BRCA2, EPCAM (3'UTR), MLH1, MSH2, MSH6, PMS2*	2
*BRCA* plus MMR genes	KSA	*BRCA1, BRCA2 MLH1, MSH2, MSH6* and *PMS2*	2
*BRCA* plus HBOC	Genetica	*BRCA1, BRCA2, ATM, BARD1, BRIP1, CDH1, CHEK2, EPCAM (3'UTR), MLH1, MSH2, MSH6, PALB2, PMS2, PTEN, RAD51C, RAD51D, STK11, TP53*	*BRCA* plus > 10 genes = 3
Extended breast- and ovarian cancer panel	KSA	*BRCA1, BRCA2, ATM, BARD1, BLM, BRIP1, CDH1, CHEK2, EPCAM, FAM175A, MEN1, MLH1, MRE11A, MSH2, MSH6, MUTYH, NBN, PALB2, PMS2, PTEN, RAD50, RAD51C, RAD51D, STK11, TP53, XRCC2*	3
Customized		*BRCA* plus > 10 genes	3
*Lynch syndrome*			
MMR genes	Basel	*MLH1, MSH2, MSH6* and *PMS2*	4

All cited laboratories underwent Swiss Accreditation (SAS) [56] based on the relevant international standards. The analyses were performed according to international standard and the results—positive and negative—were interpreted taking into account the limitation of used techniques. They provided the VUS’s scores using the most current classification, in particular according to the ACMG-Criteria [19] and using the support of Genome Aggregation Database gnomAD [57], ClinVar [18] as well as REVEL [58] and providing the newest literature citations.

### Interventions, comparisons and statistical analysis

First, we determined the overall prevalence of VUS and pathogenic/likely pathogenic (P/LP) variants in all the genetic tests of our patient group. Because some patients had more than one result (for instant a pathogenic variant in the *BRIP1* gene and a VUS in the *PMS2* gene), we used the multiple response function in SPSS. To determine the increase in VUS and P/LP variant prevalence with broader use of gene panels, our population was divided into 4 cohorts (Table 2).

Regarding HBOC, the group that underwent standard *BRCA* testing, referred to as cohort 1 (n = 632), was compared with patients who underwent gene panel testing (cohort 2 and 3). The difference between cohort 2 and 3 lies in the number of additional genes tested. Cohort 2 (n = 58) contains cases tested for *BRCA*1/2 plus ≤ 10 other genes, whereas those cases tested for > 10 additional genes are included in cohort 3 (n = 283). A detailed list of all panels used can be found under Additional file 1.

For the descriptive statistics we divided our database according to the suspected tumor syndrome, hereby cohorts 1–3 are compared to each other and cohort 4 (n = 84) is looked at separately. The comparison of cohorts 1–3 refers to the frequencies of VUS and P/LP variants and the relevance of the association was evaluated with χ^2^ test. A *P* value of less than 0.05 indicated statistical relevance.

In a further step, the two most frequently used panels for HBOC in our clinic, *BRCA* plus HBOC from Genetica [50] (n = 218) and the extended breast and ovarian cancer panel from KSA [51] (n = 54) were analyzed to determine in which genes VUS and P/LP variants occur most frequently.

Since the same four genes have been tested for LS since the beginning of genetic testing at the Inselspital, no control group could be determined. Therefore, we have identified the prevalence of VUS in patients tested for Lynch Syndrome (cohort 4) and their change over the years.

In addition we have examined in which genes variants most frequently occur and whether there is a difference of P/LP rates in male and female subjects, as previous MMR gene analysis have shown, that male sex is a predictor of variant [15, 59].

**Fig. 1:**
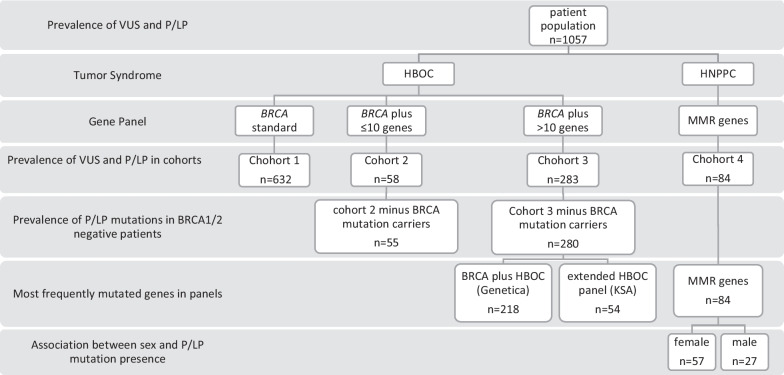
Grouping and Interventions

Figure 1 gives an overview of the grouping and interventions carried out.

## Results

### Participant characteristics

Of 1503 patients who underwent genetic testing at the Inselspital during the given period, 1057 met the inclusion criteria for our study. The other tests are not relevant for our analysis and are therefore excluded in the statistical evaluation. The ethnic background of participants is similar to the one of the general Swiss population (German 65%, French 18%, Italian 10%, Romansch 1%, other 6%) [60]

### Main findings

In 785 cases from our population (74.3%), no clinically relevant variant was found. In 147 cases (13.9%) at least one pathogenic variant and in 126 cases (11.9%) at least one VUS could be detected. In one case, we found a pathogenic variant and a VUS simultaneously and in another case two pathogenic variants were detected. 141 VUS were found in 126 different cases, as some genetic tests detected 2 or even 3 different VUS in one or several genes.

An anonymized list of all identified variants is provided in Additional file 2.

The prevalence of each test result (no variant, pathogenic variant, or VUS) in absolute numbers and percent within each cohort is shown in Table 3.

**Table 3:** Prevalence of test results in absolute numbers and percent within each cohort

Test result*cohorts Cross-tabulations^*a*^						
			Cohorts			Total
			1	2	3	
Test result	No variant	n	494	44	189	727
		% within cohorts	78.2%	75.9%	66.8%	
	Pathogenic	n	105	6	16	127
		% within cohorts	16.6%	10.3%	5.7%	
	VUS	n	38	8	90	136
		% within cohorts	6.0%	13.8%	31.8%	
Total		n	632	58	283	973
Percentages and totals are based on respondents and values may exceed 100% as patients may have multiple mutations (multiple responses possible)						
^a^Tumor syndrome = HBOC						

Test result*cohorts Cross-tabulations^*a*^				
			Cohorts	Total
			4	
Test result	No variant	Count	58	58
		% within cohorts	69.0%	
	Pathogenic	Count	22	22
		% within cohorts	26.2%	
	VUS	Count	5	5
		% within cohorts	6.0%	
Total		Count	84	84

Percentages and totals are based on respondents

^a^Tumor syndrome = Lynch

### Variants of unknown significance

In cohort 1, 38 VUS (6.0%) were found in the *BRCA1* or *BRCA2* gene. There were 8 VUS (13.8%) identified in cohort 2 and 90 VUS (31.8%) in cohort 3. As expected, VUS rates were directly proportional to the number of genes analyzed. Using the χ^2^ test, the null hypothesis can be rejected because the association between the prevalence of VUS and the increasing number of genes tested for HBOC in the cohorts is statistically significant at a level of 0.05 (*P* = 0.0E0).

**Fig. 2:**
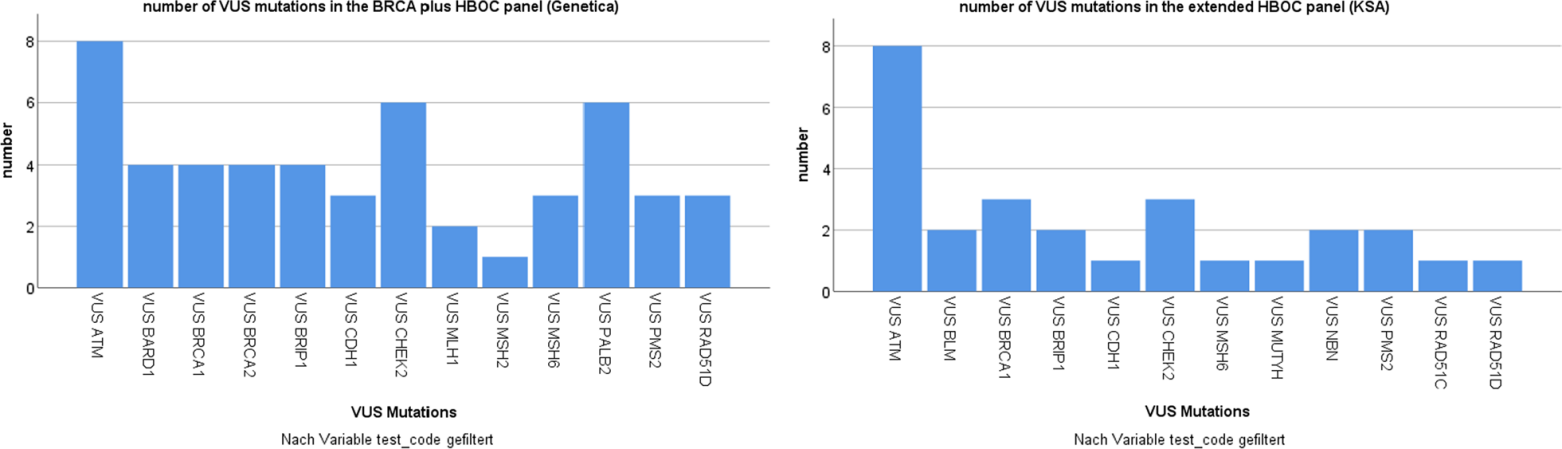
Number of VUS in the HBOC panels from the laboratories of Genetica and KSA

In the *BRCA* plus HBOC panel from Genetica containing 18 genes (Table 2), VUS were identified in 13 different genes, most frequently in *ATM* (17% of all VUS found in this Panel). Among 26 genes included in the KSA extended breast and ovarian cancer panel, *ATM* was also the gene in which the highest number of VUS was detected at 32%. In total, VUS were found in 12 different genes in this panel (Fig. 2).

In cohort 4 (Lynch Syndrome patients), 5 subjects (6.0%) were identified with a VUS. All of them were female subjects and none had an MSI-H tumor. 2 variants were found in *MLH1*, 1 in *MSH2*, 2 in *MSH6* and none in *PMS2*. Since the number of VUS found in this cohort is very small, it cannot serve as a reliable indication of whether prevalence has remained constant over the years. Only 4 VUS were found in 2019 and 1 in 2021, which made it impossible to perform a valid χ^2^ test.

### Pathogenic variants

We observed that the prevalence of P/LP variants in cohorts 1–3 relatively decrease with increasing size of the gene panel (Table 3). When *BRCA1*/2 variant carriers were excluded from cohort 2 and 3, 3 variants were detected in 55 individuals in cohort 2 (5.5%) and 11 P/LP variants in 280 individuals (3.9%) in cohort 3.

11 P/LP variants in 8 different genes could be detected in the *BRCA* plus HBOC panel from Genetica and 5 P/LP variants in 3 different genes in the extended HBOC panel from KSA.

22 (26.2%) P/LP variants were found in 21 cases of our LS population. 3 P/LP variants were found in the *MLH1* gene, 11 in *MSH2* and 8 in *MSH6*, two of them in the same subject. No deleterious variant was detected in the *PMS2* gene. 10 pathogenic variants were found in female subjects (n = 57) and 11 in male subjects (n = 27), resulting in a prevalence of pathogenic variants in female subjects of 17.5% and 40.7% in male subjects, respectively. Using χ^2^ the association between sex and prevalence of pathogenic variants in MMR genes is statistically relevant at a level of 0.05 (*P* = 0.023).

## Discussion

### Implications on our findings concerning HBOC

Consistant to other publications [61, 62], we can see a clear trend towards ordering larger panels for HBOC at our clinic over the last decade. A strong argument for panel testing is the fact that phenotypes of different cancer syndromes often overlap [53]. Breast cancer, for example, can be caused by a pathogenic variant in *BRCA1/2* genes (HBOC syndrome), but also due to a mutation in the TP53 gene (Li-Fraumeni Syndrome). Similarly, ovarian cancer can be caused by pathogenic variants in *BRCA1/2* and the MMR genes (LS). By testing multiple genes, several syndromes can be examined at once [62].

In our study, we find that the number of VUS increases while P/LP variants relatively decrease when we test a broader variety of susceptibility genes for HBOC in our population. This can be explained by the fact that in some patients screened with multigene panels, a standard *BRCA* test was performed first. Only if the result was negative (no P/LP mutation) the whole panel was analyzed. This leads to a biased low result in the number of pathogenic variants in multigene panels, as 0% had a pathogenic variant in *BRCA1*/2 in the pre-tested cases, instead of about 9–10% as in other studies [30, 33, 34]. The similar bias can be observed in the prevalence of pathogenic variants in *BRCA1*/*2* tests. Pathogenic variants turn out to be higher than expected, possibly because many standard *BRCA* tests with negative results have been expanded with a multigene panel and these cases therefore show up in cohorts 2 and 3. In particular, patients who are tested with *BRCA* plus HBOC panel from Genetica (cases of cohort 3) are often first screened for *BRCA1*/2 and the panel is directly expanded by the laboratory in case of a negative result. To avoid this bias, we specified the prevalence of pathogenic variants when *BRCA1*/2 variant carriers were excluded from cohort 2 and 3. In this case, 16 out of 347 subjects were found to have a pathogenic variant. In a similar study from Tung et al. [33] over 1700 individuals were tested with a gene panel of 11 breast cancer susceptibility genes other than *BRCA1*/2. Our result is consistent with their finding that the frequency of P/LP variants in genes susceptible to breast cancer is 4.7% in *BRCA1*/*2*-negative candidates. Our data support previous studies showing that multigene panel testing improves the identification of hereditary cancer risk in patients who meet guidelines for genetic testing for HBOC [30, 33–36].

To describe the utility of multigene panels for clinical practice, not only the increase in pathogenic variants needs to be looked at, but it is also important whether the genes are considered high or moderate risk [30] and whether they are clinically accessible or not [63]—factors we did not focus on in this study. However, another important element for the evaluation of multigene panels, which is specifically addressed in our study, is the increase in VUS rates.

The VUS rate increased from 6% when only *BRCA1/2* was tested to 13.8% when *BRCA1/2* plus ≤ 10 genes was tested and to 31.8% when *BRCA1/2* plus > 10 genes was tested, respectively. In previous studies, VUS rates have been reported to range from 6.7 to 41.7%, depending on the number of genes tested [33, 35, 64, 65]. Our results confirm this dependence between the prevalence of VUS and the number of genes tested. However, it is expected and already shown in some studies that VUS rates in less known genes will decrease over time (as they did in *BRCA1/2* [39]). The reason for this development is the ever-improving variant classification, which is progressing, among other things due to the rapid accumulation of data from family tests [35, 66] and the standardization of classification in working groups like the ClinGen Expert Panels (Variant Curation Expert Panels) [67]. The increase in the rate of VUS also correlates with the ethnicity of patients undergoing genetic testing, as a recent Stanford University study of over 1400 patients clearly showed [68]. VUS rates increased in non-white subjects from 4.4% (BRCA1/2 only) to 25–38% when 13 and 28 genes were tested, respectively. In white subjects, the prevalence of VUS increased from 3.3% only to 17% and 25%, respectively. Our study did not examine the VUS rates depending on the ethnic background of the patients. Since our patient population includes predominantly white subjects, it is reasonable to assume that the VUS rate would likely be higher in a similar study with a more diverse patient population.

The increased likelihood of an inconclusive result is an important factor in deciding between targeted testing for only one gene and the use of multigene panels, as this is obviously a challenge for both patients and physicians. Although a VUS does not change the medical course of action in a patient, growing VUS rates must not only be considered as a disadvantage of increased use of NGS gene panels. Particularly in pedigrees with an extensive cancer history, such a result may contribute to increased follow-up, taking into account the medical history, and furthermore, patients can be directly informed if a VUS result later proves to be benign or pathogenic. Of course, the usefulness of multigene panels must also take into account that with larger panels not only the number of VUS increases, but also the number of pathogenic variants found, which definitely can lead to clinical consequences.

In terms of patient management of a VUS result, Culver et al. [25] evaluated differences in cancer destress and surgical decision making between patients with BRCA negative results and patients with a VUS in *BRCA 1 or 2*, using a two year follow-up questionnaire in US patients who underwent genetic counseling due to personal or family history of breast or ovarian cancer. They concluded, that even though genetic counseling was less reassuring for participants in the VUS cohort, the result did not cause excessive surgery or exaggerated cancer distress compared to negative results.

### Implications on our findings concerning Lynch syndrome

Our results regarding LS are not very conclusive due to the small number. Nevertheless, we can give a descriptive analysis of our results and try to compare them with the existing literature. Our data show a much higher prevalence than expected, with 26.2% P/LP variants, compared with 5% in the publication by Kastrinos et al. [15]—the study on which our test criteria are based. Other than the bias due to the small number, the difference could be explained by the fact that in most cases of our study molecular testing was performed only when loss of MMR gene expression was detected in tumor tissue (exceptions were made for very young patients with a personal history strongly suggestive of LS). The number of cases tested for loss of expression was not assessed. Pathogenic variants were more common in males (40.7% of male subjects) than in females (17.5% of female subjects), suggesting that male sex is likely a predictor of variants in MMR gene analysis [15, 59]. The most frequent variants were found in the *MSH2* gene (n = 11), followed by *MSH6* (n = 8) and *MLH1* (n = 3). Other reports tend to show higher *MLH1* variant rates [69] and also databases for MMR variants list most known variants in the *MLH1* gene [17, 70]. Because our results are based on only a few pathogenic variants (n = 22), it is difficult to assess whether the low *MLH1* variant rate in our cohort is significant. None of our patients had a P/LP variant in the *PMS2* gene—consistent with the literature that variants in this gene are the least likely of all MMR genes to cause LS [17, 71].

Our VUS rate of 6% for *MLH1/MSH2/MSH6* and *PMS2* matches the prevalence of VUS in MMR genes by Eggington et al. [72]. In their poster presented at the American College of Medical Genetics and Genomics Annual Meeting, they reported the observation that VUS rates have decreased over time due to reclassification of VUS, similar to observations made for *BRCA1/2* genes. Evaluation of our annual percentage of VUS in the LS cohort should control for our assumption that the prevalence of VUS would not increase over the years because the gene panel was not expanded. However, this control proved to be inconclusive due to the very few VUS cases in this cohort.

### Limitations/strengths

Since this is a retrospective study and the cases are from multiple departments in our clinic, it is possible that individual cases were lost and not included in the study. However, a large amount of data was available to us, which increases the significance of our results. Our clinic has the largest department for oncology in the canton of Berne and thus a large access area for patients. Nevertheless, the study is not representative for the whole canton of Berne or even Switzerland, as many other (private) clinics also perform genetic testing for cancer, and we did not perform a randomized selection of patients.

Our study is also limited because the outcomes (VUS and P/LP) were compared over the years that data were collected with the most recent versions of the databases (ClinVar [18] and others) at the time of collection. Thus, for the early cases in our study, it is possible that VUS would no longer be classified as such based on current knowledge. In a future study, it would be interesting to see whether the classification of each VUS remains the same over the years or is reclassified as pathogenic/likely pathogenic or benign/likely benign and how this affects the results on the prevalence of VUS depending on the panel used.

Another limitation is that although it was investigated which genes of the HBOC panels were most frequently mutated, no distinction was made according to the indication of the test (breast/ovarian or other cancer or familial predisposition). Thus, although it is possible to estimate which genes are frequently involved in HBOC, it is not possible to say how strongly the genes are associated with the occurrence of each tumor.

## Conclusions

We found a statistically significant correlation between the rate of VUS and the increasing use of multigene panels. Our results support the use of NGS gene panels in routine diagnostics, as there is also an increase in clinically relevant variants with greater use of larger panels. With good genetic counseling before and after obtaining results, patients at risk for HBOC may benefit from larger gen panels despite increased rates of VUS.

## Supplementary Information

**Additional file 1:** List of gene panels.

**Additional file 2:** Anonymized list of identified variants.

**Additional file 3:** Anonymized raw data.

## Data Availability

The datasets used during the current study are available from the corresponding author on reasonable request.

## Abbreviations

ACMG: American College of Medical Genetics and Genomics
AMP: Association for Molecular Pathology
ENIGMA: Evidence-based Network for the Interpretation of Germline Mutation Alleles
HBOC: Hereditary breast and ovarian cancer
HNPPC: Hereditary non-polyposis colorectal cancer
IARC: International Agency for Research on Cancer
InSiGHT: International Society for Gastrointestinal Hereditary Tumours
LS: Lynch syndrome
MMR: Mismatch repair
NCCN: National Comprehensive Cancer Network
NGS: Next generation sequencing
P/LP: Pathogenic/likely pathogenic mutation
SAKK: Schweizerische Arbeitsgemeinschaft für Klinische Krebsforschung
UKMO: Universitätsklinik für Medizinische Onkologie
VUS: Variant of unknown significance
