# Supplementary material for: Occurrence of variants of unknown clinical significance in genetic testing for hereditary breast and ovarian cancer syndrome and Lynch syndrome: a literature review and analytical observational retrospective cohort study
Source: BMC Med Genomics. 2023 Jan 16;16:7. doi: 10.1186/s12920-023-01437-7 (PMC9843935; doi:10.1186/s12920-023-01437-7)
Supplement: Supplementary file 1 — Additional file 1: List of gene panels. [file 12920_2023_1437_MOESM1_ESM.docx]

**Additional file 1: list of gene panels**

| **Panel** | **List of genes** | **N (how often the panel was ordered in our population)** |
| --- | --- | --- |
| **Hereditary breast and ovarian cancer** |  |  |
| BRCA standard | *BRCA1 and BRCA2* | 632 |
| BRCA plus Mamma/  Breast cancer panel | *BRCA1, BRCA2, ATM, CDH1, CHEK2, PALB2, PTEN, STK11, TP53* | 20 |
| BRCA plus Ovar  Ovarian cancer panel | *BRCA1, BRCA2, BRIP1, EPCAM (3’UTR), MLH1, MSH2, MSH6, PMS2, RAD51C, RAD51D* | 1 |
| BRCA plus HBOC | *BRCA1, BRCA2, ATM, BARD1, BRIP1, CDH1, CHEK2, EPCAM (3'UTR), MLH1, MSH2, MSH6, PALB2, PMS2, PTEN, RAD51C, RAD51D, STK11, TP53* | 218 |
| Extended breast- and  ovarian cancer panel | *BRCA1, BRCA2, ATM, BARD1, BLM, BRIP1, CDH1, CHEK2, EPCAM, FAM175A, MEN1, MLH1, MRE11A, MSH2, MSH6, MUTYH, NBN, PALB2, PMS2, PTEN, RAD50, RAD51C, RAD51D, STK11, TP53, XRCC2* | 54 |
| BRCA plus Lynch | *BRCA1, BRCA2, EPCAM (3'UTR), MLH1, MSH2, MSH6, PMS2* | 5 |
| BRCA plus MMR genes | *BRCA1, BRCA2 MLH1, MSH2, MSH6 und PMS2* | 5 |
| Customized BRCA plus ≤10 genes |  |  |
|  | *BRCA1, BRCA2, CDH1* | 2 |
|  | *BRCA1, BRCA2, RAD51C* | 3 |
|  | *BRCA1, BRCA2, TP53* | 5 |
|  | *BRCA1, BRCA2, ATM* | 1 |
|  | *BRCA1, BRCA2, MEN1* | 1 |
|  | *BRCA1, BRCA2, PALB2* | 2 |
|  | *BRCA1, BRCA2, CDK4, CDKN2A* | 1 |
|  | *BRCA1, BRCA2, ATM, PALB2, TP53* | 1 |
|  | *BRCA1, BRCA2, ATM, CDH1, CHEK2, PALB2, STK11, TP53* | 1 |
|  | *BRCA1, BRCA2, ATM, CHEK2, PALB2, PTEN, STK11, TP53* | 1 |
|  | *BRCA1, BRCA2, CDKN2A, CDKN2B, CDK4, TP53* | 1 |
|  | *BRCA1, BRCA2, CDKN2A, CDKN2B, CDK4, TP53, WRN* | 1 |
|  | *BRCA1, BRCA2, BARD1, BRP1, MLH1, NBN, PMS2, PTEN, RAD51D, TP53* | 1 |
| BRCA plus Lynch, CDH1 | *BRCA1, BRCA2, CDH1, EPCAM (3'UTR), MLH1, MSH2, MSH6, PMS2* | 1 |
| BRCA plus MMR, FAP | *BRCA1, BRCA2, APC, MUTYH, MLH1, MSH2, MSH6, PMS2* | 1 |
| BRCA plus MMR, ATM, CHEK2, PALB2 | *BRCA1, BRCA2, ATM, CHEK2, MLH1, MSH2, MSH6, PALB2, PSM2* | 2 |
|  | *BRCA1, BRCA2, APC, ATM, CDH1, MSH2, MSH6, MUTYH, PALB2, PTEN, STK11, TP53* | 1 |
| BRCA Standard und Melanom | *BRCA1, BRCA2, BAP1, BRCA2, CDK4, CDKN2A, MITF, TERT (Promotor)* | 1 |
| Customized BRCA plus >10 genes |  |  |
|  | *BRCA1, BRCA2, ATM, BARD1, BRIP1, CHEK2, MLH1, MSH2, MSH6, PALB2, PMS2, PTEN, RAD51C, RAD51D, STK11, TP53* | 1 |
|  | *BRCA1, BRCA2, ATM, BRIP1, CHEK2, EPCAM (3'UTR), MLH1, MSH2, MSH6, PALB2, PMS2, PTEN, RAD51C, RAD51D, STK11, TP53* | 1 |
| BRCA plus HBOC, NBN | *BRCA1, BRCA2, ATM, BARD1, BRIP1, CDH1, CHEK2, EPCAM (3'UTR), MLH1, MSH2, MSH6, PALB2, PMS2, PTEN, RAD51C, RAD51D, STK11, TP53, NBN* | 1 |
| BRCA plus HBOC, EGFR, RECQL5 | *BRCA1, BRCA2, ATM, BARD1, BRIP1, CDH1, CHEK2, EPCAM (3'UTR), MLH1, MSH2, MSH6, PALB2, PMS2, PTEN, RAD51C, RAD51D, STK11, TP53, EGFR, RECQL5* | 1 |
| BRCA Standard und colon cancer, ATM, CDH1, PALB2 | *BRCA1, BRCA2, APC, BMPR1A, CHEK2, EPCAM (3'UTR), GREM1, MLH1, MSH2, MSH6, MUTYH, PMS2, POLD1, POLE, PTEN, SMAD4, STK11, TP53, ATM, CDH1, PALB2* | 1 |
|  | *APC, BMPR1A, BRCA1, BRCA2, CELA3B, CFTR, CHEK2, CPA1, CTRC, EPCAM, GREM1, KRT8, MLH1, MSH2, MSH6, MUTYH, PMS2, POLD1, POLE, PRSS1, PTEN, SMAD4, SPINK1, STK11, TP53, TRPV6* | 1 |
|  | *BRCA1, BRCA2, APC, ATM, BARD1, BMPR1A, BRIP1, CDH1, CHEK2, EPCAM, GREM1, MLH1, MSH2, MSH6, MUTYH, NBN, PALB2, PRKAR1A, PMS2, POLD1, POLE, PTEN, PMS2, RAD51C, RAD51D, SMAD4, STK11, TP53* | 1 |
| BRCA plus HBOC, colon cancer and melanoma | *BRCA1, BRCA2, ATM, BARD1, BRIP1, CDH1, CHEK2, EPCAM (3'UTR), MLH1, MSH2, MSH6, PALB2, PMS2, PTEN, RAD51C, RAD51D, STK11, TP53, APC, BMPR1A, GREM1, MUTYH, POLD1, POLE, SMAD4, BAP1, CDK4, CDKN2A, MITF, TERT (Promotor)* | 1 |
|  | *BRCA1, BRCA2, ATM, CHECK2, EPCAM, HOXB13, KIT, MLH1, MSH2, MSH6, PALB2, PDGFRA, PMS2, RAD51C, RAD51D, SDHA, SDHAF2, SDHB, SDHC, SDHD* | 1 |
|  | *BRCA1, BRCA2, BLM, BRIP1, ERCC4, FANCA, FANCB, FANCC, FANCD2, FANCE, FANCF, FANCG, FANCI, FANCL, FANCM, MAD2L2, PALB2, RAD51, RAD51C, SLX4, TOP3A, UBE2T, XRCC2* | 1 |
|  | *BRCA1, BRCA2, BLM, BRIP1, ERCC4, FANCA, FANCB, FANCC, FANCD2, FANCE, FANCF, FANCG, FANCI, FANCL, PALB2, SLX4, TOB3, UBE2T, MAD2L2, RAD51C, FANCM, XRCC2* | 1 |
| **Lynch syndrome** |  |  |
| MMR genes | *MLH1, MSH2, MSH6 and PMS2* | 84 |
